# Primary care engagement is associated with increased pharmacotherapy prescribing for alcohol use disorder (AUD)

**DOI:** 10.1186/s13722-019-0147-3

**Published:** 2019-05-01

**Authors:** Paul J. Joudrey, Mat Kladney, Chinazo O. Cunningham, Marcus A. Bachhuber

**Affiliations:** 1 Present Address: VA Connecticut Healthcare System, West Haven Campus, 950 Campbell Ave, West Haven, CT 06516 USA; 2 National Clinician Scholars Program, Yale School of Medicine, 333 Cedar Street, Sterling Hall of Medicine I-456, PO Box 208088, New Haven, CT 06520 USA; 3 Division of General Internal Medicine, Montefiore Medical Center/Albert Einstein College of Medicine, 111 E. 210th Street, Bronx, NY 10467 USA

**Keywords:** Alcohol use disorder, Pharmacotherapy, Primary care, Engagement, Pharmacoepidemiology, Health care services, Health care utilization

## Abstract

**Background:**

Primary care provider skills such as screening, longitudinal monitoring, and medication management are generalizable to prescribing alcohol use disorder (AUD) pharmacotherapy. The association between primary care engagement (i.e., longitudinal utilization of primary care services) and prescribing of AUD pharmacotherapy is unknown.

**Methods:**

We examined a 5-year (2010–2014) retrospective cohort of patients with AUD, 18 years and older, at an urban academic medical center in the Bronx, NY, USA. Our main exposure was level of primary care engagement (no primary care, limited primary care, and engaged with primary care) and our outcome was any AUD pharmacotherapy prescription within 2 years of AUD diagnosis. Using multivariable logistic regression, we examined the association between primary care engagement and pharmacotherapy prescribing, accounting for demographic and clinical factors.

**Results:**

Of 21,159 adults (28.9% female) with AUD, 2.1% (n = 449) were prescribed pharmacotherapy. After adjusting for confounders, the probability of receiving an AUD pharmacotherapy prescription for patients with no primary care was 1.61% (95% CI 1.39, 1.84). The probability of AUD pharmacotherapy prescribing was 2.56% (95% CI 2.06, 3.06) for patients with limited primary care and 2.89% (95% CI 2.44, 3.34%) for patients engaged with primary care.

**Conclusions:**

The percentage of AUD patients prescribed AUD pharmacotherapy was low; however, primary care engagement was associated with a higher, but modest, probability of receiving a prescription. Efforts to increase primary care engagement among patients with AUD may translate into increased AUD pharmacotherapy prescribing; however, strategies to increase prescribing across health care settings are needed.

**Electronic supplementary material:**

The online version of this article (10.1186/s13722-019-0147-3) contains supplementary material, which is available to authorized users.

## Background

Alcohol use disorder (AUD) is a significant cause of morbidity and mortality but remains under-diagnosed and undertreated. In the United States, 68.5 million adults develop an AUD during their lifetime [1] and 88,000 alcohol-related deaths occur annually [2]. In 2010, high risk alcohol use cost the United States $249 billion [3]. Despite the heavy human and economic toll of AUD, only 71.1% of adults receiving outpatient medical care reported receiving any assessment of alcohol use, and only 2.9% of those with alcohol abuse and 7.0% of those with alcohol dependence were offered information about treatment at the time of diagnosis [4]. Furthermore, only 8.3% of adults with AUD actually receive treatment for unhealthy alcohol use at a specialized treatment center [5].

AUD pharmacotherapy (acamprosate, naltrexone, disulfiram, and topiramate) is efficacious in improving consumption outcomes (prevention of return to any drinking or return to heavy drinking) and broadly recommended by clinical guidelines [6, 7]. Historically, AUD pharmacotherapy prescribing occurred in specialty settings; however integration of AUD care into primary care is increasingly recommended [8–10]. Primary care settings provide an opportunity for AUD treatment because of primary care providers’ ability to diagnose AUD both through screening and through detecting medical conditions or symptoms caused or exacerbated by alcohol use [11, 12]. Further, primary care provider skills, such as longitudinal monitoring and medication management, and organizational strategies can improve chronic disease outcomes and are readily generalized to AUD pharmacotherapy [13, 14].

Chronic diseases like AUD require assessment and longitudinal follow-up from a regular source of care. Primary care engagement represents a series of visits with a primary care provider [15] and is associated with improved health outcomes for several chronic diseases [16]. Greater patient engagement in primary care may make this setting superior for delivering treatment than specialty AUD treatment settings [17]. Given the difficulty of connecting AUD patients to specialty care, improving primary care engagement among AUD patients and increasing pharmacotherapy prescribing in primary care may be a promising strategy for improving AUD outcomes [18]. Despite this potential, the link between primary care engagement and prescribing of AUD pharmacotherapy remains unclear. To address this gap in knowledge, we conducted a retrospective cohort study of patients in a large urban health care delivery system. During the study period, this health care delivery system did not have organizational guidelines or programs to support the prescribing of AUD pharmacotherapy.

## Methods

### Setting and data sources

Montefiore Medical Center is an academic medical center and integrated health care delivery system in the Bronx, NY. In addition to 4 hospitals (over 80,000 total inpatient admissions annually) and 4 emergency departments (over 300,000 total visits annually), Montefiore provides primary and specialty care through a network of outpatient clinics with over 3 million visits annually. We conducted a retrospective cohort study using all de-identified patient, emergency department visit, outpatient visit, inpatient hospitalization, problem list, and prescription data from the Montefiore electronic health record between January 1, 2010 and December 31, 2016. The Montefiore Medical Center/Albert Einstein College of Medicine Institutional Review Board approved this study.

### Study population

We included all patients: (1) 18 years and older, (2) with an AUD diagnosis, defined as an International Classification of Diseases, 9th Edition, Clinical Modification (ICD-9-CM), code or free-text problem list entry indicating an AUD (Additional file 1: Appendix 1) entered between January 1, 2010 and December 31, 2014. Given evidence that AUD remains under-diagnosed among patients with regular contact with the health care system [4], we included problem list entries “alcohol consumption binge drinking” and “alcohol consumption heavy” as evidence of AUD diagnosis, while also tracking how many entered our cohort with either of these problem list entries. A diagnosis code indicating AUD in remission did not qualify for cohort entry. We excluded patients if they were prescribed AUD pharmacotherapy within the 6 months prior to the AUD diagnosis. Patients entered the cohort at the time of AUD diagnosis and we extracted data on each patient for 2 years after this index date.

### Main exposure: level of primary care engagement

Our main exposure was level of primary care engagement within 2 years of AUD diagnosis. Similar to previous research [19], we coded primary care engagement as a three-level categorical variable: no primary care, limited primary care, or engaged with primary care. We defined “no primary care” as no visit to a primary care facility (i.e., internal or family medicine primary care clinic). We defined “primary care engagement” as one visit to a primary care facility (among 21 primary care facilities within the Montefiore Medical Center network) within 1 year of AUD diagnosis and at least one additional visit at the same facility between 90 and 365 days after the first visit. We defined “limited primary care” as one or more visits to a primary care facility but no additional visits to the same primary care facility between 90 and 365 days after the first primary care visit.

### Main outcome: prescription of AUD pharmacotherapy

For our primary outcome, we defined AUD pharmacotherapy prescribing as at least one instance of a provider prescription for acamprosate, naltrexone, topiramate, or disulfiram within 2 years of AUD diagnosis. This approach includes all medications approved by the Food and Drug Administration plus topiramate, which we included given the evidence for efficacy within a 2014 meta-analysis [6]. We examined the percentage of patients who had any AUD pharmacotherapy prescription ordered, and for each individual medication.

### Demographic and clinical characteristics

For eligible patients, we extracted demographic and clinical characteristics, and health care utilization data. For demographic characteristics, we extracted: age, sex (male, female), race/ethnicity (Hispanic/Latino of any race, White non-Hispanic, Black non-Hispanic, and other/unknown), and insurance status (private, public, self-pay/unknown). For clinical characteristics, we extracted: year of cohort entry (i.e., year of AUD diagnosis), Charlson comorbidity index based on ICD-9-CM codes 1 year prior to AUD diagnosis [20, 21], and presence of a psychiatric comorbidity within 1 year prior to AUD diagnosis (yes, no; based on an ICD-9-CM code or problem list entry indicating depression, anxiety, schizophrenia, or bipolar disorder, Additional file 1: Appendix 2). For health care utilization, we extracted the number of: emergency department visits, inpatient hospitalizations, outpatient psychiatric visits, and outpatient substance use disorder treatment visits within 2 years of AUD diagnosis.

### Statistical analysis

First, by level of primary care engagement (no primary care, limited primary care, and engaged with primary care), we compared demographic and clinical characteristics, and health care utilization. For categorical variables we used Pearson Chi square test, and for continuous variables we used Kruskal–Wallis tests.

Next, to assess the association between level of primary care engagement (main exposure) and AUD pharmacotherapy prescribing (main outcome), we used a multivariable logistic regression model. We included age, sex, race/ethnicity, year of cohort entry, insurance status, Charlson score, psychiatric comorbidity, emergency department visits, inpatient hospitalizations, and outpatient psychiatric and substance use specialty visits in the model because all were associated with the primary outcome during bivariate testing with a *P* value < 0.2. We did not find evidence of collinearity between variables in our final model. We present logistic regression results as predictive margins and adjusted differences based on recycled prediction with all analyses completed in Stata 13 (StataCorp, College Station, TX).

### Sensitivity analysis

We conducted two sensitivity analyses to determine the robustness of our findings. To account for alternative indications for prescribing topiramate, we removed patients with epilepsy and migraine headache syndromes (based on ICD9-CM codes or problem list entries, Additional file 1: Appendix 3) noted within 1 year prior to cohort entry. To account for a more narrow definition of AUD, we repeated our analysis while excluding patients with either “alcohol consumption binge drinking” and “alcohol consumption heavy” problem list entries.

## Results

Of 21,159 adult AUD patients in our cohort, almost half were between the ages of 45–64 (45.4%), and the majority were male (71.1%; Table 1). Non-Hispanic Black (35.7%) was the most common race/ethnicity. Only 2.1% of patients were prescribed AUD pharmacotherapy. Of those individuals prescribed AUD pharmacotherapy, topiramate (0.9%, n = 196) and naltrexone (0.8%, n = 161) were the most prescribed medications followed by acamprosate (0.4%, n = 82) and disulfiram (0.2%, n = 42).

**Table 1:** Demographic, clinical, and health care utilization characteristics of patients with alcohol use disorder in a large urban academic medical center (n = 21,159)

Characteristic	All patients (n = 21,159)	No primary care^c^ (n = 12,086;57.1%)	Limited primary care^d^ (n = 3583; 16.9%)	Engaged with primary care^e^ (n = 5490; 26.0%)	*p* value
Age, n (%)					< 0.001
18–29	3242 (15.3)	2203 (18.2)	540 (15.1)	499 (9.1)	
30–44	4962 (23.5)	3179 (26.3)	825 (23.0)	958 (17.5)	
45–64	9614 (45.4)	5063 (41.9)	1661 (46.4)	2890 (52.6)	
65 +	3341 (15.8)	1641 (13.6)	557 (15.6)	1143 (20.8)	
Female sex, n (%)	6104 (28.9)	3027 (25.1)	1083 (30.2)	1994 (36.3)	< 0.001
Race/ethnicity, n (%)					< 0.001
Hispanic	2028 (9.6)	647 (5.4)	444 (12.4)	937 (17.1)	
Non-hispanic white	3402 (16.1)	2235 (18.5)	504 (14.1)	663 (12.1)	
Non-hispanic black	7554 (35.7)	4060 (33.6)	1329 (37.1)	2165 (39.4)	
Any other^a^ or undetermined	8175 (38.6)	5144 (42.6)	1306 (36.5)	1725 (31.4)	
Insurance status					< 0.001
Public	13,130 (62.1)	7598 (62.9)	2293 (64.0)	3239 (59.0)	
Private	7452 (35.2)	4017 (33.2)	1248 (34.8)	2187 (39.8)	
Self-pay or undetermined	577 (2.7)	471 (3.9)	42 (1.2)	64 (1.2)	
Charlson score, n (%)					< 0.001
0	11,353 (53.7)	7340 (60.7)	1837 (51.3)	2176 (39.6)	
1–2	5800 (27.4)	2976 (24.6)	960 (26.8)	1864 (34.0)	
3+	4006 (18.9)	1770 (14.7)	786 (21.9)	1450 (26.4)	
Year of cohort entry, n (%)					< 0.001
2010	5148 (24.3)	2898 (24.0)	832 (23.2)	1418 (25.8)	
2011	3942 (18.6)	2379 (19.7)	632 (17.6)	931 (17.0)	
2012	3821 (18.1)	2183 (18.1)	636 (17.8)	1002 (18.3)	
2013	4106 (19.4)	2297 (19.0)	684 (19.1)	1125 (20.5)	
2014	4142 (19.6)	2329 (19.3)	799 (22.3)	1014 (18.5)	
Psychiatric comorbidity^b^, n (%)	5860 (27.7)	3209 (26.6)	987 (27.6)	1664 (30.3)	< 0.001
Emergency visits, median (IQR)	1 (1, 3)	1 (1, 3)	2 (1, 4)	1 (0, 4)	< 0.001
Inpatient admissions, median (IQR)	1 (0, 2)	1 (0, 1)	1 (0, 2)	1 (0, 2)	< 0.001
Outpatient psychiatry visits, median (IQR)	0 (0, 0)	0 (0, 0)	0 (0, 0)	0 (0, 0)	< 0.001
Outpatient substance use treatment visits, median (IQR)	0 (0, 0)	0 (0, 0)	0 (0, 0)	0 (0, 0)	< 0.713

^a^Includes Asian/Pacific Islander, Native American/Alaskan Native, and more than one race or missing race

^b^Includes depression, anxiety disorder, bipolar disorder, and schizophrenia within 1 year prior to cohort entry

^c^No primary care visits within 2 years of cohort entry

^d^Any number of primary care visits within 2 years of cohort entry that did not meet criteria for engaged with primary care

^e^At least two primary care facility visits, the first within 1 year of cohort entry and the second between 90 and 365 days after

During the 2 years after AUD diagnosis, 57.1% of AUD patients had no primary care, 16.9% had limited primary care, and 26.0% were engaged with primary care. Compared to patients with no primary care, patients with limited primary care and patients engaged with primary care were significantly different in all demographic, clinical, and health care utilization characteristics.

In the no primary care, limited primary care, and engaged with primary care groups, the unadjusted percentages of patients receiving an AUD pharmacotherapy prescription were 1.6%, 2.7%, and 3.0%, respectively. In adjusted analyses, the probability of patients with no primary care receiving an AUD pharmacotherapy prescription was 1.61% (95% CI 1.39, 1.84). The probability of patients with limited primary care receiving an AUD pharmacotherapy prescription was 2.56% (95% CI 2.06, 3.06) and 2.89% (95% CI 2.44, 3.34%) for patients engaged with primary care (Table 2).

**Table 2:** Factors associated with receiving a prescription for alcohol use disorder pharmacotherapy within 2 years of diagnosis in a large urban academic medical center (n = 21,159)

Variable	Probability, % (95% CI)^a^	Adjusted difference, percentage points^b^ (95% CI)	*p* value
Age			
18–29	1.29 (0.90, 1.69)	Ref.	–
30–44	2.83 (2.37, 3.28)	1.53 (0.94, 2.12)	< 0.001
45–64	2.38 (2.07, 2.68)	1.08 (0.58, 1.59)	< 0.001
65+	0.92 (0.57, 1.27)	-0.37 (-0.90, 0.17)	0.181
Sex			
Male	2.01 (1.78, 2.24)	Ref.	–
Female	2.35 (2.00, 2.71)	0.34 (− 0.09, 0.77)	0.109
Race/ethnicity			
Non-hispanic white	2.86 (2.29, 3.42)	Ref.	–
Hispanic	2.71 (2.10, 3.32)	− 0.15 (− 0.99, 0.69)	0.730
Non-hispanic black	1.65 (1.37, 1.93)	− 1.20 (− 1.84, − 0.57)	< 0.001
Any other^a^ or undetermined race	2.07 (1.75, 2.39)	− 0.79 (− 1.44, − 0.14)	0.011
Insurance status			
Public	2.16 (1.92, 2.40)	Ref.	–
Private	2.06 (1.72, 2.41)	− 0.09 (− 0.53, 0.34)	0.679
Self-pay or undetermined	1.89 (0.71, 3.08)	− 0.27 (− 1.48, 0.95)	0.685
Charlson score			
0	2.41 (2.09, 2.72)	Ref.	–
1–2	2.41 (2.04, 2.78)	0.00 (-0.50, 0.50)	0.998
3+	1.16 (0.86, 1.47)	-1.24 (-1.71, -0.78)	< 0.001
Year to cohort entry			
2010	1.60 (1.28, 1.92)	Ref.	–
2011	2.06 (1.62, 2.50)	0.46 (− 0.08, 1.01)	0.091
2012	1.95 (1.53, 2.38)	0.36 (− 0.18, 0.89)	0.188
2013	2.52 (2.05, 2.99)	0.92 (0.35, 1.49)	0.001
2014	2.70 (2.20, 3.20)	1.10 (0.50, 1.69)	< 0.001
Psychiatric comorbidity			
No	1.52 (1.32, 1.73)	Ref.	–
Yes	3.34 (2.89, 3.78)	1.81 (1.31, 2.31)	< 0.001
Emergency visits			
0 visits	2.16 (1.90, 2.41)	Ref.	
1 visits	2.15 (1.92, 2.38)	− 0.01 (− 0.05, 0.03)	0.686
Inpatient admissions			
0 admissions	1.78 (1.58, 1.98)	Ref.	
1 admission	1.98 (1.79, 2.16)	0.22 (0.13, 0.30)	< 0.001
Outpatient psychiatry visits			
0 visits	1.96 (1.78, 2.15)	Ref.	
5 visits	2.15 (1.95, 2.35)	0.19 (0.14, 0.24)	< 0.001
Outpatient substance use treatment visits			
0 visits	2.07 (1.88, 2.26)	Ref.	
5 visits	2.65 (2.32, 2.97)	0.52 (0.31, 0.73)	< 0.001
Level of primary care engagement			
No primary care	1.61 (1.39, 1.84)	Ref.	
Limited primary care	2.56 (2.06, 3.06)	0.95 (0.40, 1.50)	< 0.001
Engaged with primary care	2.89 (2.44, 3.34)	1.28 (0.76, 1.79)	< 0.001

^a^Refers to the adjusted probability of receiving a prescription for alcohol use disorder pharmacotherapy, obtained from a multivariable logistic regression model adjusting for each factor presented in the table and exposure of interest. See Additional file 1: Appendix 4 for further details

^b^Refers to the difference in the probability of receiving a prescription for alcohol use disorder pharmacotherapy, after adjusting for all other factors described in the table

In our sensitivity analysis, compared with patients with no primary care, the probability of receiving an AUD pharmacotherapy prescription remained higher for patients with limited primary care (adjusted difference: 0.71 percentage points, 95% CI 0.18, 1.24) and for patients engaged with primary care (adjusted difference: 1.21 percentage points, 95% CI 0.69, 1.72). In our original analysis, 63 study subjects entered the cohort with a problem list entry “alcohol consumption binge drinking” or “alcohol consumption heavy,” representing less than 0.01% of the total study sample. Removing these individuals from the study did not change the overall portion (2.1%) of patients who received AUD pharmacotherapy.

## Discussion

In our study examining the relationship between primary care engagement and AUD pharmacotherapy prescribing, primary care engagement was associated with a higher, but modest, probability of receiving an AUD pharmacotherapy prescription. However, the overall percentage of AUD patients prescribed AUD pharmacotherapy was less than 3% even among those engaged with primary care, suggesting broad underutilization.

Our findings are consistent with previous work showing that most adults with an AUD do not receive evidence-based treatment [1, 4, 5]. Further, the low numbers of patients prescribed AUD pharmacotherapy in our study are similar to Veterans Health Administration facilities data and studies of national prescription rates [22–27]. Our study extends these low prescribing numbers to patients engaged with primary care services. Barriers to AUD pharmacotherapy prescribing include a lack of provider and patient knowledge about pharmacotherapy, formulary restrictions, provider comfort and perceptions of pharmacotherapy effectiveness [28, 29]. Our findings represent prescribing within a health care delivery system without guidelines or programs to address these barriers and improve prescribing. Evidenced-based primary care implementation strategies addressing these barriers are needed to achieve higher rates of AUD pharmacotherapy prescribing in primary care.

Our results show an association between primary care engagement and AUD pharmacotherapy prescribing. Further research should determine if improving AUD patient primary care engagement increases pharmacotherapy prescribing. Patients prescribed AUD pharmacotherapy may be more likely to engage with primary care services; therefore, changing the level of engagement among additional AUD patients may not necessarily increase prescribing. However, strategies to improve primary care engagement in other chronic diseases have improved medication initiation and adherence [30, 31]. The management of AUD as a chronic disease may similarly benefit from improved primary care engagement.

While increased efforts to engage AUD patients in primary care could translate into higher rates of prescribing, nearly 60% of our cohort had no contact with primary care services at our institution. Strategies to link patients from emergency or inpatient units to AUD pharmacotherapy and primary care services for longitudinal management are necessary to reach a broad population of AUD patients. In a recent study, a standard protocol for treatment of hospitalized AUD patients resulted in increased AUD pharmacotherapy prescribing at discharge, which supports the feasibility and efficacy of inpatient AUD pharmacotherapy strategies, but the subsequent level of primary care engagement among such patients remains unclear [32].

This study has several limitations. First, while our absolute AUD pharmacotherapy rates are similar to other studies, our results may not be generalizable to other settings which differ from a large urban academic medical center. Second, because the determination of level of primary care engagement required following patients for the entire duration of the follow up period (2 years), we cannot determine if the prescribing of pharmacotherapy preceded or followed primary care engagement and can only determine an association between primary care engagement and prescribing of AUD pharmacotherapy. Third, we could not account for health care utilization and prescribing of AUD pharmacotherapy at outside institutions. Fourth, because we used ICD-9-CM diagnosis codes and problem list entries, some patients may be misclassified as having AUD or not having AUD and we could not precisely measure AUD severity. Most guidelines recommend consideration of AUD pharmacotherapy for patients with moderate or severe disease; therefore, our estimates of AUD pharmacotherapy may be conservative. Finally, we focused on a measure of initial prescription of AUD pharmacotherapy and did not assess adherence (prescriptions dispensed) or duration of therapy.

## Conclusions

Overall, while primary care engagement was associated with a higher, but modest, probability of receiving a prescription for AUD pharmacotherapy, few patients were prescribed AUD pharmacotherapy. Strategies to greatly increase overall rates of AUD pharmacotherapy in health care settings are needed to improve the proportion of patients receiving evidence-based care. Primary care engagement may be one potential strategy to increase prescribing of AUD pharmacotherapy.

## Additional file

**Additional file 1.** Supplementary materials. Alcohol use disorder, psychiatric, seizure, and migraine diagnostic criteria used for this study and an explanation of predictive margins.

## Abbreviations

AUD: alcohol use disorder
ICD-9-CM: International Classification of Diseases, 9th Edition, Clinical Modification
