# Supplementary material for: Primary care engagement is associated with increased pharmacotherapy prescribing for alcohol use disorder (AUD)
Source: Addict Sci Clin Pract. 2019 May 1;14:19. doi: 10.1186/s13722-019-0147-3 (PMC6492411; doi:10.1186/s13722-019-0147-3)
Supplement: Supplementary file 1 — Additional file 1. Supplementary materials. Alcohol use disorder, psychiatric, seizure, and migraine diagnostic criteria used for this study and an explanation of predictive margins. [file 13722_2019_147_MOESM1_ESM.docx]

**Supplementary Materials**

1. Appendix

1.1 AUD diagnosis criteria

1.1.1 Problem List Entries for AUD diagnosis

Acute alcohol abuse

Alcohol abuse

Alcohol consumption binge drinking

Alcohol consumption heavy

Alcohol Dependence

Alcohol withdrawal

Alcohol withdrawal seizure

Alcohol withdrawal syndrome

Alcoholism

Chronic alcohol abuse

Alcohol abuse continuous use

Episodic chronic alcoholism

Alcohol detoxification

1.1.2 ICD9 Codes for AUD diagnosis

291 Alcohol induced mental disorders

Delirium tremens

Alcohol-induced persisting amnestic disorder

Alcohol-induced persisting dementia

Alcohol induced psychotic disorder

Pathological alcohol intox

Alcohol-induced psychotic disorder

Other specified alcoholic psychosis

Alcohol withdrawal

Alcohol induced sleep disorders

Other special alcohol-induced mental disorder

Unspecified alcohol-induced mental disorder

303 Alcohol dependence syndrome

Acute alcohol intox continuous

Alcohol Dep NOS

Alcohol Dep Continuous

Alcohol Dep Episodic

305 Alcohol abuse

Alcohol Abuse unspecified

Alcohol Abuse continuous

Alcohol Abuse episodic

2.2 Psychiatric comorbidity diagnosis criteria

2.2.1 Depression ICD9

293.83 Mood disorder in conditions classified

296.20 Depression psychosis-unspec

296.21 Depression psychosis mild

296.22 Depression psychosis mod

296.23 Depression psychosis severe

296.24 Depression psychosis severe with psych

296.25 Depression psychosis part remission

296.30 Recurrent depression psychosis unspecified

296.31 Recurrent depression psychosis mild

296.32 Recurrent depression psychosis moderate

296.33 Recurrent depression psychosis severe

296.34 Recurrent depression psychosis psychotic

296.35 Recurrent depression psychosis partial remission

296.82 Atypical depressive disorder

296.99 other specified episodic mood disorder

300.4 Dysthymic disorder

311 Depressive disorder

2.2.2 Depression problem list

Adjustment disorder with depressive mood

Agitated depression

Atypical depression

Depression

Depressive disorder

Depressive psychosis

Depressive psychosis mild single episode

Depressive psychosis severe single episode

Depressive state

Episode of moderate major depression

Major depression

Major depression single episode

Mild major depression single epsiode

Moderate depressive psychosis

Moderate recurrent depressive psychosis

Moderate recurrent major depression

Moderate single major depressive episode

Psychotic depression

Reactive depressive psychosis

Recurrent  depressive psychosis

Recurrent major depression, mild episode

Recurrent severe major depression

Severe depressive psychosis

Severe recurrent depression w psychosis

Severe recurrent  depressive psychosis

Major depressive single episode severe with psychosis

Major depression single episode severe without psychosis

Severe major depression, single episode, without psychosis

2.2.3 Anxiety ICD9

293.84 Anxiety disorder in conditions classified

300.00 Anxiety state nos

300.01 Panic disorder w/o agophobia

300.02 Generalized anxiety disorder

300.09 Anxiety state nec

300.10 Hysteria nos

300.20 Phobia nos

300.21 Agoraphobia with panic disorder

300.22 Agoraphobia without panic disorder

300.23 Social phobia

300.29 Other isolated or specific phobias

300.3 Obsessive compulsive disorder

300.5 Neurasthenia

300.89 Other somatoform disorders

308.0 Stress reaction emotional

308.1 Stress reaction, fugue

308.2 Stress reaction, psychomotor

308.3 Acute stress reaction nec

308.4 Stress reaction, mixed disorder

308.9 Acute stress reaction nos

309.81 Post traumatic stress disorder

313.0 Overanxious disorder

2.2.4 Anxiety Problem List

Acute anxiety

Adjustment reaction with anxiety

Agoraphobia

Agoraphobia with panic

Anticipatory anxiety

Anxiety

Anxiety disorder

Anxiety disorder due to brain injury

Anxiety neurosis

Anxiety reaction

Anxiety state

Chronic  anxiety

Claustrophobia

Generalized anxiety disorder

Job related stress

Light phobia

Mixed disorder stress reaction

Noise phobia

Panic attack

Panic disorder

Panic state

Phobia

Post traumatic stress reaction

Separation anxiety

Social phobia

Post traumatic stress disorder

Situational anxiety

Simple phobia

2.2.5 Schizophrenia ICD9

295 Schizophrenia disorders

295.0 Simple schizophrenia

295.00 Simple schizophrenia unspec

295.01 Simple schizophrenia subchronic

295.02 Simple schizophrenia chronic

295.04 Simple schizo chronic exacerbation

295.2 Catatonic schizophrenia

295.3 Paranoid schizophrenia

295.4 AC schizophrenia episode

295.5 Latent schizophrenia

295.6 Residual schizophrenia

295.6 Schizophrenic disorder, residual

295.7 Schizoaffective type

295.8 Schizophrenia nec

295.9 Schizophrenia nos

2.2.6 Schizophrenia Problem List

Acute exacerb chronic hebephrenic schizophrenia

Acute exacerb chronic paranoid schizophrenia

Acute exacerb chronic schizoaffective disorder

Acute exacerbation chronic latent schizophrenia

Acute exacerbation of chronic schizophrenia

Chronic hebephrenic schizophrenia

Chronic latent schizophrenia

Chronic paranoid schizophrenia

Chronic schizophrenia

Chronic undifferentiated schizophrenia

Disorganized schizophrenia

Hebephrenic schizophrenia

Latent schizophrenia

Paranoid schizophrenia

Residual schizophrenia

Schizoaffective disorder

Schizoaffective psychosis

Schizoaffective type schizophrenia

Schizophrenia

Undifferentiated schizophrenia

Schizoaffective disorder bipolar type

2.2.7 Bipolar ICD9

296.00-05 Bipolar I single manic episode

296.40-45 Bipolar affective manic

296.50-55 Bipolar affect, depressed

296.60-65 Bipolar affective, mixed

296.7 Bipolar I recent episode unspecified

296.89 Other unspecified bipolar disorders

2.2.8 Bipolar Problem List

Bipolar affective disorder

Bipolar affective disorder with mania

Bipolar affective psychosis

Bipolar disorder

Bipolar disorder type II

Bipolar disorder w moderate depression

Bipolar disorder with depression

Bipolar disorder with moderate mania

Bipolar disorder with severe depression

Bipolar disorder with severe mania

Bipolar mixed affective disorder, moderate

Depressed bipolar affective disorder

Manic type bipolar affective disorder

Mixed bipolar affective disorder

Mixed type bipolar affective disorder

Bipolar 1 disorder mixed mild

Bipolar disease in pregnancy

3.3 Migraine and seizure diagnosis criteria

3.3.1 Migraine ICD9 Codes

346 Migraine

346.0 Classical migraine

346.1 Common Migraine

346.2 Variants of Migraine

346.8 Other forms of migraine

346.9 Migraine unspecified

3.3.2 Migraine Problem List

Atypical migraine

Basilar migraine

Classic migraine

Common migraine

Hemiplegic migraine

Intractable migraine

Migraine

Migraine headache

Ocular migraine

Ophthalmoplegic migraine

Intractable classic migraine

Intractable common migraine

Migraine headache with aura

Refractory migraine

3.3.3 Seizure disorders ICD9

345 Epilepsy

345.0 Gen non convulsive epilepsy

345.1 Gen convulsive epilepsy

345.4 Psychomotor epilepsy

345.41 LCL epilepsy

345.5 Partial epilepsy nec

345.8 Epilepsy nec

345.9 Epilepsy nos

3.3.4 Seizure disorder Problem List

Atonic epilepsy

Atypical absence epilepsy

Atypical absence epilepsy intractable

Benign rolandic epilepsy

Cognitive dysfunction with epilepsy

Complex partial epilepsy

Epilepsy

Gelastic epilepsy

Generalized nonconvulsive epilepsy

Generalized tonic clonic epilepsy

Intractable complex partial epilepsy

Intractable epilepsy

Intractable gen tonic epilepsy

Intractable myoclonic epilepsy

Intractable partial epilepsy

Intractable partial epilepsy with impairment

Intractable simple partial epilepsy

Kojewnikoffs epilepsy

Myoclonic epilepsy

Myoclonus epilepsy

Partial epilepsy without impairment

Partial epilepsy with impairment

Photogenic epilepsy

Simple partial epilepsy

Tonic epilepsy

Epilepsy complicating pregnancy

4. Explanation of predictive margins in Table 2

We have chosen to present the results of our multivariable logistic regression using predictive margins rather than traditional odds ratios. Predictive margins have the advantage of reporting the results of the model as an estimate of the probability of the outcome among a population with a given characteristic while controlling for the other covariates in the model. Communicating the results in terms of probability rather than odds makes the results more comparable and understandable. For example, in Table 2, the “Outpatient psychiatry visits” can be interpreted in the following manor. Among patients who had zero outpatient psychiatry visits during the preceding year, when all other covariates are set to their reference values, the probability of receiving AUD pharmacotherapy was 1.96%. For patients who had 5 outpatient psychiatry visits during the preceding year, when all other covariates are set to the reference values, the probability of receiving AUD pharmacotherapy was 2.15%. The change in probability (adjusted difference) between someone with 0 outpatient psychiatry visits and 5 outpatient psychiatry visits was 0.19%. We chose to use 5 outpatient psychiatry visits simply because the change in probability (adjusted difference) between zero and one outpatient psychiatry visits was exceedingly small and difficult to report. We therefore present the change in probability in terms of the change in probability of the outcome when comparing a population with 0 vs 5 outpatient psychiatry visits.
